# Factors impacting loneliness in patients with serious life-limiting illness in the Emergency Medicine Palliative Care Access (EMPallA) study

**DOI:** 10.1186/s12904-025-01699-1

**Published:** 2025-03-08

**Authors:** Brendan Maloney, Mara Flannery, Jason J. Bischof, Kaitlyn Van Allen, Oluwaseun Adeyemi, Keith S. Goldfeld, Allison M. Cuthel, Alex Chang, Corita R. Grudzen

**Affiliations:** 1 SUNY Upstate Medical University, Syracuse, NY, United States; 2 Ronald O. Perelman Department of Emergency Medicine, New York University Grossman School of Medicine, 227 East 30th Street, New York, NY 10016 USA; 3 Department of Emergency Medicine, The Ohio State University Wexner Medical Center, Columbus, OH USA; 4 Department of Population Health, New York University Grossman School of Medicine, New York, NY USA; 5 College of Science, Northeastern University, Boston, MA USA; 6 Division of Supportive and Acute Care Services, Memorial Sloan Kettering Cancer Center, New York, NY USA

**Keywords:** Advanced cancer, End-stage organ failure, Functional decline, Geriatrics, Palliative care, Patient-reported outcomes, Loneliness

## Abstract

**Background:**

Loneliness is a quality-of-life (QoL) concern for patients facing serious, life-limiting illnesses. Discerning risk factors of loneliness in palliative care patients allows providers to take preventative action and develop holistic treatment plans.

**Methods:**

A planned sub-study of patients who completed the previously developed Three-Item Loneliness Scale upon enrollment into the multicenter, randomized clinical trial Emergency Medicine Palliative Care Access (EMPallA) with the objective of investigating the association of multimorbidity with loneliness in patients with late-stage illnesses. The EMPallA study included patients who were at least 50 years old and diagnosed with at least one end-stage illness (advanced cancer, advanced congestive heart failure (CHF), end-stage renal disease (ESRD), or advanced chronic obstructive pulmonary disease (COPD)).

**Results:**

We analyzed 1,212 surveys using a mixed-effects logistic regression model. Our findings suggest those with a single illness are less likely to be lonely than those with multimorbidity (odds ratio [OR] = 0.5, 95% CI 0.3 to 0.8). Additionally, older age was associated with less loneliness (OR comparing age by 10-year increments is 0.7 [95% CI: 0.6 to 0.9]), after adjusting for disease type, education level, race, sex, immigrant status, having a caregiver, COVID-19 period, language, and site geographic location.

**Conclusions:**

Patients suffering from multimorbidity self-report being “very lonely” more often than patients with a single advanced illness; furthermore, advanced illness patients who were middle-aged (versus elderly) were 25% more likely to report being “very lonely.”

**Trial registration:**

Clinicaltrials.gov identifier: NCT03325985. Registered October 30, 2017.

## Background

Palliative care, a patient-centered model of care, employs an interdisciplinary team to improve the quality of life (QoL) of patients of all ages living with advanced illnesses [1]. The interdisciplinary team of healthcare professionals, social workers, mental health professionals, and chaplains collaborate in working towards establishing healthy mental wellbeing and social networks, using a variety of techniques [2]. Establishing a healthy mental wellbeing through palliative care encompasses addressing feelings of loneliness which afflicts patients with serious life-limiting illnesses [2].

Wenger et al. defines loneliness, a common health-related issue in seriously ill patients, as the “subjective feelings associated with isolation, whether that be less than desired contact or no contact at all with others [3].” Patients suffering from advanced illnesses often experience loneliness, increasing pain, and an increasing awareness of death as their health deteriorates [2]. These sentiments are fueled by a loss of identity and sense of self in isolation, expedited by growing physical limitations as their terminal illnesses advance [4]. Critical towards improving QoL, treating patients’ loneliness may limit further declines in mental or physical health. In many cases, loneliness exacerbates underlying conditions and can act as a source of anxiety, depression, and other mental health illnesses [5].

Patients with advanced stage illness are also more likely to report decreased physical activity [6]. Post-diagnosis, chronic pain often forces patients to limit social interactions, whether due to treatment-related exhaustion, or general reductions in mobility. The toll this exhaustion has on physical activity only progresses with their illness, as current palliative care emphasizes intensifying therapeutic and pharmacologic interventions to accompany an advancing illness [6]. Balancing their own treatment plans and current symptoms causes an isolative effect due to a shift in the patients focus from their social connections to their own treatment management, and they may experience dwindling social networks found in family members, friends, employment, and healthcare professionals [2, 7].

Many patients associate loneliness with feelings of shame, and thus, conceal their true feelings to avoid stigmatization [8]. The tendency of patients to conceal their loneliness emphasizes the importance of developing awareness towards the risk factors of loneliness. Present literature describes an association between loneliness and a diagnosis of a life-limiting illnesses (e.g. cancer [9], chronic obstructive pulmonary disease [10], and congestive heart failure [11]). However, there is limited literature focused on the association between feelings of loneliness and multiple serious life-limiting illnesses in the palliative care setting. This study, therefore, aims to examine the association between comorbid illnesses in patients with serious life-limiting illnesses and loneliness. Further, this study’s results will improve the awareness of palliative care providers to not only care for patients experiencing loneliness, but also identify patients at high risk of developing loneliness. Identifying patients with loneliness earlier on can allow for interventions such as integration of socializing in their treatment plans or the incorporation of a therapist into the healthcare team.

## Methods

### Study design and population

This is a planned secondary analysis of data persons enrolled in Emergency Medicine Palliative Care Access (EMPallA), a comparative effectiveness, multi-center randomized controlled trial testing two forms of palliative care delivery for persons living with serious illness following emergency room (ED) discharge: nurse-led telephonic care versus specialty outpatient palliative care [12]. Patients were enrolled between April 2018 and June 2022 at 18 EDs within 15 healthcare systems in nine states. Enrollment occurred either at discharge in the ED or in patients’ homes via telephone. The larger trial was approved by all sites’ respective institutional review boards and funded by the Patient-Centered Outcomes Research Institute (PCORI) (ClinicialTrials.gov: Identifier NCT03325985).

### Measures

Upon enrollment, patients completed a sociodemographic questionnaire along with a baseline Three-Item Loneliness Scale survey [13].

### Three-item loneliness scale

The Three-Item Loneliness Scale survey is a validated previously published survey derived from the Los Angeles 13-Item Loneliness Scale (UCLA-13). It comprises three questions in a three-point Likert scale with a Cronbach alpha score ranging from 0.89 to 0.94, indicating good internal reliability [14]. Each answer choice was given a score of one (“hardly ever”), two (“some of the time”), or three (“often”), resulting in a minimum score of three and a maximum score of nine. For this analysis, we re-categorized patients’ scores such that patients reporting a score of six or less were considered “not very lonely,” and those scoring seven or greater were described as “very lonely [15–17].”

### Inclusion and exclusion criteria

Patient eligibility criteria included being at least 50 years old at time of enrollment, speaking either English or Spanish, residing in a pre-defined geographic area, and being diagnosed with one of the following advanced illnesses: advanced-stage cancer (metastatic solid tumor), congestive heart failure (CHF) (New York Heart Association (NYHA) class III or IV), chronic obstructive pulmonary disease (COPD) (GOLD stage III or IV, forced expiratory volume (FEV1) < 50%, or oxygen dependent), or end-stage renal disease (ESRD) (either dialysis dependent or a glomerular filtration rate (GFR) less than 15 ml/min/m2). Exclusion criteria included having two or more palliative care outpatient visits within the prior six months, hospice care within the prior six months, residence in a long-term care facility (skilled nursing facility or nursing home) during time of enrollment, any diagnosis of dementia, or a lack of a working telephone [12].

### Analysis

All covariates were pre-specified based on a literature review of their potential to predict loneliness [18–24]. We employed a mixed-effects logistic regression model with a binomial distribution to identify predictors of loneliness status [25]. The overall Three-Item Loneliness Scale score was dichotomized as “not very lonely” versus “very lonely”. The model adjusted for baseline loneliness, age, sex, race, ethnicity, functional status, illness, education, presence of a caregiver, COVID-19 period, marital status, income, language, site location, religion, and immigrant status. Disease type was categorized into six categories upon enrollment: “Cancer Only”, “CHF Only”, “COPD Only”, “ESRD Only”, “Cancer with Comorbidity” or “Non-cancer with Comorbidity”. For analysis, “Cancer with Comorbidity” and “Non-cancer with Comorbidity” were combined into a single category (“Has Comorbidities”), while the other four categories were grouped as “Does not have comorbidities.” Patient multimorbidity was defined as the presence of both advanced-stage cancer and end-stage organ failure or multiple end-stage organ failures. For discussion purposes only, age was categorized into three groups: 50–59 years of age, 60–69 years of age, and 70 + years of age. However, for analysis, patient age was treated as a continuous variable. All analyses were completed using R, Version 4.1.2 (R Foundation for Statistical Computing).

## Results

A total of 1,284 baseline surveys were completed, and 1,212 were analyzed. Seventy-two surveys contained incomplete data and were excluded. The following missing data were observed: educational background (3%, *n* = 40), race (1%, *n* = 17), immigrant status (< 1%, *n* = 11), Three-Item Loneliness Scale score (< 1%, *n* = 9), or presence of a caregiver (< 1%, *n* = 8) information.

### Patient demographics

The mean age of the study population was 67 (SD 10). Approximately 48% (*n* = 583) of patients were male, and 43% (*n* = 522) identified as non-white, and 11% (*n* = 129) identified as Hispanic or Latino. Approximately 21% (*n* = 256) required at least considerable functional assistance. A majority of patients (62%, *n* = 751) had completed education beyond high school. Approximately 37% (*n* = 446) had been diagnosed with advanced-stage cancer, and 10% (*n* = 122) reported having multiple advanced illnesses (cancer or end-stage organ failure), which is defined as multimorbidity for this analysis. Additionally, 61% (*n* = 737) reported having a caregiver (Table 1).

**Table 1:** Sociodemographic characteristics of the sample (*n* = 1,212)

Variable	*n* (%)
Age (years)	
Mean (SD)	67 (10)
Sex	
Female	629 (52)
Male	583 (48)
Race	
White	690 (57)
Black	386 (32)
Multi-Race	21 (2)
Other	115 (10)
Ethnicity (Missing = 17)	
Hispanic or Latino/a	129 (11)
Not Hispanic or Latino/a	1066 (89)
Functional status (Missing = 1)	
Normal activity	333 (27)
Cares for self, unable to do normal activity	250 (21)
Requires occasional assistance	372 (31)
Requires considerable assistance	185 (15)
Disabled	71 (6)
Illness	
Cancer only	423 (35)
CHF only	263 (22)
ESRD only	229 (19)
COPD only	175 (14)
Non-cancer, has multiple illnesses	99 (8)
Cancer, has multiple illnesses	23 (2)
Highest educational level completed	
< HS Degree	153 (13)
HS Degree	308 (25)
Some College/AA degree	365 (30)
College Degree or >	386 (32)
Caregiver	
Yes	737 (61)
No	475 (39)
COVID-19 period	
Pre	444 (37)
Post	768 (63)
Marital Status (Missing = 10)	
Married or living with a partner	525 (44)
Separated or divorced	250 (21)
Never married	246 (20)
Widowed	167 (14)
Other	14 (1)
Income (Missing = 212)	
< $25k	457 (46)
$25k - $49k	203 (20)
$50k - $99k	192 (19)
$100k or >	148 (15)
Primary language	
English	1178 (97)
Spanish	34 (3)
Urban	
Yes	720 (59)
No	492 (41)
Religion (Missing = 30)	
Is religious	839 (71)
Is not religious	343 (29)
Born in United States	
Yes	1072 (88)
No	140 (12)
FACT-G (Missing = 5)	
Mean (SD)	65 (18)
ESAS-r (Missing = 5)	
Mean (SD)	40 (13)
Three-Item Loneliness Scale	
Not Very Lonely	946 (78)
Very Lonely	266 (22)

The total number of patients for each variable are shown here along with the percentage of total patients that are within each variable. For Age, FACT-G, and ESAS-r categories, the mean value of each is reported along with their standard deviation

*Note* SD: standard deviation, CHF: congestive heart failure, COPD: chronic obstructive pulmonary disease, ESRD: end-stage renal disease, HS: high school, AA: associate of arts, FACT-G: Functional Assessment of Cancer Therapy – General, ESAS-r: Edmonton Symptom Assessment Scale - revised

### Outcomes

Approximately 226 (21%) study participants living with only one advanced illness (*n* = 1,090) were categorized as being “very lonely.” For participants living with two or more advanced illnesses (*n* = 127), 40 (33%) reported being “very lonely.” Roughly 27% of patients between the ages of 50 and 59 reported being “very lonely”, compared to 21% of patients 60 to 69, and 18% of patients aged 70 years or older.

After adjusting for patient-level characteristics, patients with only one illness were less likely to be “very lonely” than patients with multimorbidity (Odds ratio [OR] = 0.5, 95% CI 0.3 to 0.8) (Table 2; significant relationships are reported in bold). Overall, patients who were older (OR = 0.97, 95% CI 0.96 to 0.99), had a caregiver (OR = 0.5, 95% CI 0.4 to 0.7), were diagnosed with cancer (OR = 0.4, 95% CI 0.3 to 0.5), had more education (OR = 0.7, 95% CI 0.51 to 0.94), were male (OR = 0.7, 95% CI 0.6 to 0.99), were enrolled at a suburban site (OR = 0.6, 95% CI 0.4 to 0.8), and had only one illness versus multimorbidity (OR = 0.5, 95% CI 0.3 to 0.8) were less likely to report feeling “very lonely” (Table 2).

**Table 2:** Logistic regression model results

Independent variables	Dependent variable	
	OR (95% CIs)	P
Age	**0.97 (0.96**,** 0.99)**	< 0.001
More Education	**0.70 (0.51**,** 0.94)**	0.019
Has a Caregiver	**0.50 (0.37**,** 0.67)**	< 0.001
Enrolled during COVID-19 period	0.87 (0.64, 1.18)	0.371
Male	**0.74 (0.55**,** 0.99)**	0.043
Suburban Residence	**0.57 (0.38**,** 0.82)**	0.001
Immigrant Status	0.78 (0.46, 1.27)	0.327
No comorbidities	**0.53 (0.34**,** 0.82)**	0.004
Has Cancer	**0.37 (0.26**,** 0.53)**	< 0.001
Spanish-Speaking	0.83 (0.31, 2.04)	0.704
Non-White	0.79 (0.57, 1.09)	0.146

The logistic regression output is shown here. The first value indicates the odds ratio, and the values within parentheses indicates the 95% confidence interval associated with each variable. Reported education levels were categorized into “more education” (self-reported at least some college) vs. “less education” (self-reported at most a high school diploma). Enrollment timeframe was categorized into “enrolled during COVID-19 period” vs. “enrolled before COVID-19 period”. Residence was categorized into “suburban” vs. “urban”. Primary language was categorized as “Spanish-speaking” vs. “English-speaking”. Statistically significant relationships are shown in bold.

## Discussion

Within this study, pooled responses to the Three-Item Loneliness Scale survey revealed that patients diagnosed with multimorbidity experience increased loneliness, as indicated by a higher proportion of “very lonely” responses when compared with those with a single advanced diagnosis. Due to patients’ perceived and real burden associated with multimorbidity, they may feel increasingly isolated from their friends and family [26]. Multimorbidity diagnoses may lead patients to feel that they are unable to speak openly and honestly about their treatments and illnesses due to their complexity and the stigma associated with each diagnosis, leading to difficulties in fostering meaningful relationships. Recently diagnosed patients often struggle to manage their new treatment plans, further exacerbating the strain put on their social wellbeing especially when the patient may already struggle with managing existing care for previously diagnosed advanced illnesses [27]. It is critical for palliative care to account for patients’ loneliness and catering their treatment plan to their loneliness level, which can be inferred from their comorbid status.

Understanding these unique burdens afflicting each patient can lead to the development of more personalized treatment plans as part of Advance care planning (ACP). Previous literature reveals that those experiencing severe loneliness were less likely to be enrolled in ACP, whether in the form of end-of-life discussions, advanced directives, or the establishment of a durable power of attorney [28]. Predictive standards determining the relationship between a patient’s comorbidity status and their likelihood of experiencing loneliness can therefore facilitate targeted, personalized, and early ACP interventions.

As loneliness is commonly experienced in patients with serious, advanced illnesses [3], early identification of the risk factors associated with loneliness is key when a patient is referred to palliative care. Multiple advanced stage illness diagnoses can serve as a call to action for palliative care providers to adapt treatment plans with patients who may be experiencing loneliness or have a declining social network. Targeted interventions can follow initial consultations, with outpatient services like Hanna et al.’s ‘Day Hospices’ demonstrating notable improvements in peer-support communities available to patients, and a decrease in caregiver burden [29].

Of note, patients in our oldest age bracket were less likely to report feelings of loneliness compared to patients aged 50–59 years old. As patients’ age, they are more likely to require assistance with their activities of daily living leading to the enlistment of a caregiver [30]. Patients with an enlisted caregiver have more social interaction compared to other patients without regular caregiver interaction. This regular interaction can help to alleviate their feelings of loneliness, explaining the findings of older patients being less likely to report severe feelings of loneliness. This line of reasoning is supported by our results showing that patients with an assigned caregiver were less likely to report feelings of loneliness compared to those without a designated caregiver. Men were also found to be less likely to report feelings of loneliness compared to women which is found to be aligned with current literature supporting men being less likely to admit feelings of loneliness compared to women [31]. These results support this finding even in a population of patients undergoing palliative care.

### Practice and research implications

The findings have several implications for practice. For patients undergoing palliative care, the addition of a social worker to their treatment team will aid in patient’s pain management skills. Social workers can dedicate time to training patients in techniques to manage their pain which not only aid in pain management but also aid in decreasing patients’ feelings of loneliness [32]. Social workers also integrate caregivers into patients’ treatment which further works towards managing patients’ loneliness. Palliative care delivered through an interdisciplinary team manages both the patient’s physical symptoms and psychosocial wellbeing.

Treatment plans focused on improving their social networks and reducing patients’ symptoms of loneliness will improve their overall QoL. Taking extra precautions for patients with multimorbidity by preparing further support for these patients along with proper counseling for organizing their medical treatments can prevent feelings of loneliness from developing.

In a rapidly advancing field, it is key for palliative care providers to have a comprehensive understanding of certain risk factors that patients diagnosed with advanced illnesses can exhibit. Early palliative care interventions are critical when attempting to improve or maintain a patient’s QoL. Providers understanding the risk factors for loneliness and instituting consistent screening with valid and reliable instruments can be decisive in relieving patients’ feelings of loneliness and advancing research efforts on the topic [33].

### Limitations

Several limitations exist within this sub-study. The study population only enrolled patients who are at least 50 years of age, excluding patients who are younger than 50 but are also candidates for palliative care. Additionally, patients who speak English or Spanish and fit specific geographic residence criteria were eligible for enrollment, limiting the diversity of the study population. This sub-study only measures patient loneliness that is reported at baseline values and fails to capture other objective measures of loneliness. Future studies should incorporate multiple objective measures of loneliness to further enrich the construct of social isolation. Additionally, the variables employed in the regression analysis are limited to variables available in the parent study and future research should employ additional variables rooted in a conceptual model [33]. Efforts identifying techniques or methods to help decrease patients’ feelings of loneliness would serve to further enhance the literature and standard of care surrounding this topic.

## Conclusion

Patients diagnosed with multimorbidity (advanced cancer, CHF, COPD, and/or ESRD) are more likely to experience symptoms of loneliness than patients diagnosed with a single advanced illness. Acknowledging that these patients are at a higher risk of experiencing symptoms of loneliness allows palliative care experts to take preventative measures in their treatment plans to alleviate these symptoms and improve the patient’s QoL.

## Data Availability

All data generated and/or analysed during the current study or sub-study are available from the corresponding author on reasonable request.

## Abbreviations

QoL: Quality of Life
EMPallA: Emergency Medicine Palliative Care Access
ED: Emergency Department
PCORI: Patient-Centered Outcomes Research Institute
COPD: Chronic Obstructive Pulmonary Disease
FEV1: Forced Expiratory Value
GOLD: Global Initiative for Chronic Obstructive Lung Disease
CHF: Congestive Heart Failure
NYHA: New York Heart Association
ESRD: End-Stage Renal Disease
GFR: Glomerular Filtration Rate
IQR: Inter-Quartile Range
CI: Confidence Interval
VR: Virtual Reality
